# Successful Management of Complicated Uterine Displacement Caused by Unilateral Incarceration of the Bicornuate Uterus

**DOI:** 10.1155/2019/3205610

**Published:** 2019-03-07

**Authors:** Nozomi Ouchi, Yoshimitsu Kuwabara, Mirei Yonezawa, Ryuhei Kurashina, Tomoko Ichikawa, Rintaro Sawa, Toshiyuki Takeshita

**Affiliations:** Department of Obstetrics and Gynecology, Nippon Medical School Hospital, 1-1-5 Sendagi, Bunkyo-ku, Tokyo, Japan

## Abstract

Uterine incarceration is a serious complication of pregnancy, in which the gravid uterus becomes trapped in the posterior pelvis. When labor occurs, delivery does not progress, and the uterus may rupture. Therefore, preoperative diagnosis of uterine incarceration is important, and a caesarian section is indispensable except when the polarity of the uterus can be successfully restored. We report the case of a 35-year-old primipara with a complication of a bicornuate uterus who became pregnant after in vitro fertilization and embryo transfer. No abnormality was observed on regular checkups until the second trimester. At 28 weeks' gestation, the uterine cervix revealed marked dislocation, and, at 31 weeks, magnetic resonance imaging (MRI) revealed uterine cervix elongation and left horn incarceration. At 37 weeks' gestation, an elective cesarean section was performed. On laparotomy, the uterus was found to be markedly dislocated, and distended blood vessels were observed on the surface. Ultrasound examination was performed directly on the uterine wall to decide the incision site. After delivery of the baby, manual repositioning of the uterus revealed the unique concurrent clockwise rotation and retro-vertical deflection. Thus, we concluded that incarceration accompanied by a bicornuate uterus can cause complicated uterine displacement, and preoperative MRI and intraoperative ultrasound examination are useful for managing this condition.

## 1. Introduction

Uterine incarceration is a serious complication of pregnancy, reported to occur at a frequency of 1/3000-1/10000 per pregnancy [1–3]. In this condition, the gravid uterus becomes trapped in the posterior pelvis, which sometimes causes pelvic discomfort, lower abdominal or back pain, and dysuria [4]. However, asymptomatic uterine incarceration is considered extremely rare. Only 28 cases of this condition, which were initially diagnosed in the third trimester, were identified in the past 147 years according to a systematic search of English articles in EMBASE and PubMed [5].

When labor occurs, delivery does not proceed and uterine rupture may occur [6]. Therefore, preoperative diagnosis is important and caesarian section is indispensable except when the polarity of the uterus can be successfully restored. Deciding the uterine incision site is the most important, because inappropriate incision into the extended cervical canal, accompanied by incarceration, may cause cervical canal amputation and/or massive bleeding. We report a case of successfully managed uterine incarceration which showed complex uterine displacement, with retroflexion, as well as marked clockwise and vertical rotation, by trapping of the unilateral horn of the bicornuate uterus.

## 2. Case

A 35-year-old primipara visited our hospital because of a history of two miscarriages. Diagnostic screening tests revealed a retroverted bicornuate uterus and deficiency of* factor XII*, which may have been related to the previous recurrent abortion. Owing to secondary sterility, her third pregnancy was the result of in vitro fertilization and embryo transfer, and low-dose aspirin therapy was started after embryo transfer. In the first trimester, a gestational sac was detected on the right side of the uterine horn. At 18 weeks and 4 days of gestation, no abnormality in the fetal or placental position was detected by transvaginal ultrasonography.

**Figure 1:**
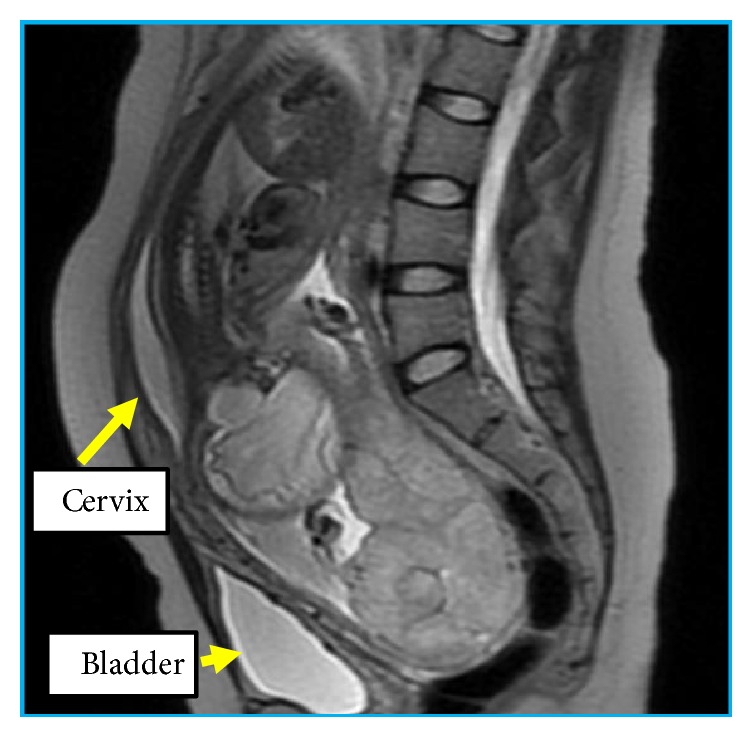
Magnetic resonance imaging scan at 31 weeks and 2 days of gestation.

**Figure 2:**
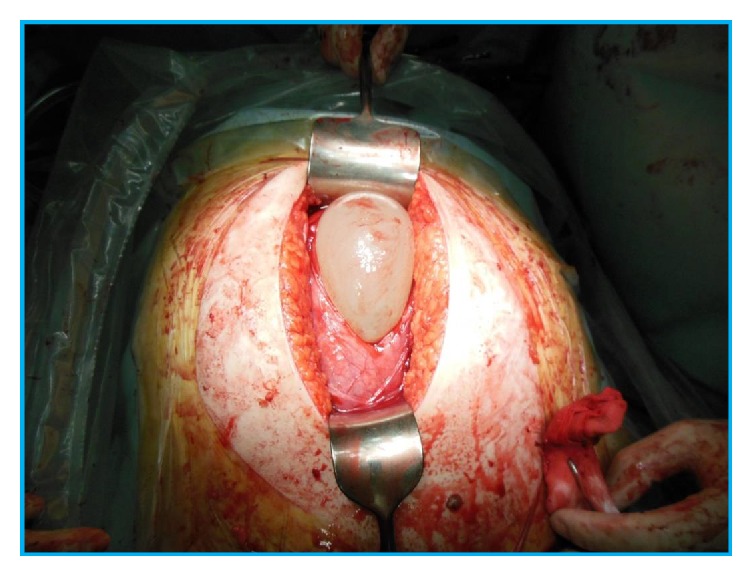
The uterus is cut vertically.

**Figure 3:**
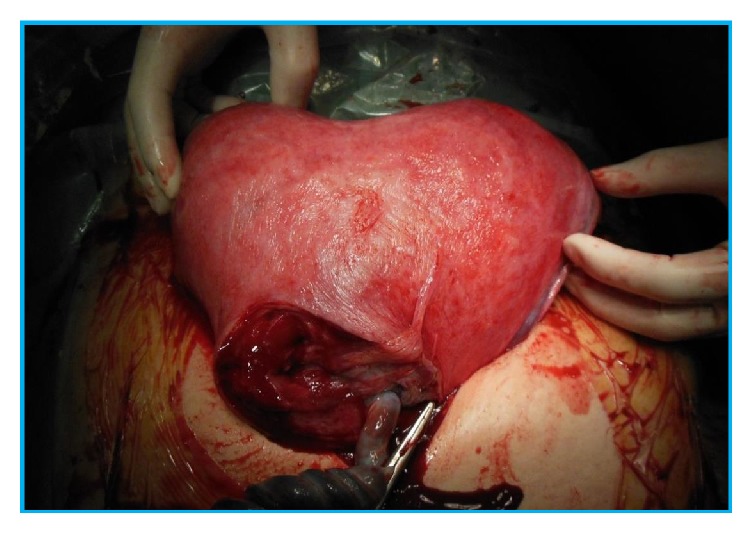
The uterus is repositioned after delivery of the baby.

At 28 weeks and 4 days of gestation, marked dislocation of the uterine cervix was suggested by speculum and internal genital examinations. On transvaginal ultrasound examination, the uterine cervix was difficult to detect, whereas the placenta was located on the maternal dorsal side, mimicking the placenta previa. At 31 weeks and 2 days of gestation, MRI was performed to assess the positions of the uterus, placenta, and fetus. The left side of the uterine horn containing a major part of the placenta showed marked retroversion and was trapped in the Douglas cavity. The uterine cervix showed elongation and was located behind the bladder. The fetus was on both sides of the uterine horn, showing a transverse position (Figure 1). This was considered a unique case of unilateral incarceration of the bicornuate uterus showing complicated uterine retroversion. She did not notice any characteristic symptoms such as abdominal pain or urinary retention throughout the gestational period. At 37 weeks and 3 days of gestation, an elective cesarean section was performed. On laparotomy, the uterus was markedly rotated, and distended blood vessels were observed on the surface. Ultrasound examination was performed directly on the uterine wall to check the fetal and placental positions. A vertical incision of the recognized uterine wall under the umbilical part of the abdominal wall was made (Figure 2), and a live male infant, weighing 2500 grams, was born. The uterus was repositioned manually, and a 45° clockwise and 90° retrovertical rotation was confirmed. Furthermore, the site of the uterine wall incision was located in the lower uterine segment transversely, which is the usual position for a normal cesarean section (Figure 3), and the distended blood vessels were identified as the uterine artery and vein.

## 3. Discussion

Uterine incarceration is known to be caused by uterine myoma, endometriosis, adhesion after intraperitoneal inflammation, or malformation of the uterus [2, 7]. Although there are a few reports on uterus incarceration complicated by a bicornuate uterus [8–10], the clinical characteristics of such cases remain unknown.

This case revealed a risk of complex displacement of the bicornuate uterus accompanied by concurrent rotation and deflection when a unilateral corn is trapped in the Douglas pouch. This is a novel point that has not been suggested in previous reports showing bilateral incarcerated corns of the bicornuate uterus. Because of marked displacement of the uterus accompanied by distended vessels on the surface, it appeared difficult to decide the uterine incision site just by setting it higher than usual, which is the standard strategy for cesarean section of an incarcerated uterus.

To manage this condition successfully, a two-stage setting of preoperative MRI and intraoperative ultrasound examination was helpful. The effectiveness of MRI in the diagnosis of uterine incarceration has been previously reported [1, 11]. In this case, the possibility of placenta previa or a low-lying placenta was accurately excluded by MRI, and it could also be predicted that only a unilateral corn was incarcerated in the Douglas pouch. On the other hand, it was difficult to make a surgical design for the uterine incision site at this point. From the laparotomy findings, it was still difficult to assess the anatomical displacement of the uterus accurately, and the appropriate incision site was finally determined by intraoperative ultrasound examination. To our knowledge, there are no reports concerning the usefulness of an intraoperative direct ultrasound examination at the time of caesarean section in the English literature. However, in some cases of anterior low-lying placenta or placenta previa, we have recognized that a direct intraoperative ultrasound examination is useful in determining the appropriate incision site to avoid placental parenchyma, and it is considered beneficial for reducing maternal bleeding and fetal blood loss. By taking advantage of this technique, the concurrent evaluation of macroscopic findings of the uterine surface and ultrasonographic findings of the intrauterine cavity enabled us to avoid amputation of the extended cervix and determine a safe incision site where the placenta does not exist.

Repositioning of the displaced uterus after delivery of the baby revealed that the site of the uterine wall incision was located in the usual lower uterine segment and that the distended blood vessels that were located in front of the operative field were arteriovenous and originally located in the parametrium of the uterus.

In conclusion, in the case of uterine incarceration complicated by a bicornuate uterus, it is important to remember that severe uterine displacement caused by complex rotation may exist. Preoperative MRI and intraoperative ultrasound examination are useful to manage this condition, especially to decide on the appropriate site for uterine incision during cesarean section.
